# Genome-Wide Analysis of Acute Endurance Exercise-Induced Translational Regulation in Mouse Skeletal Muscle

**DOI:** 10.1371/journal.pone.0148311

**Published:** 2016-02-04

**Authors:** Hiroaki Sako, Koichi Yada, Katsuhiko Suzuki

**Affiliations:** 1 Graduate School of Sport Sciences, Waseda University, Saitama, 359–1192, Japan; 2 Faculty of Sport Sciences, Waseda University, Saitama, 359–1192, Japan; University of Birmingham, UNITED KINGDOM

## Abstract

Exercise dynamically changes skeletal muscle protein synthesis to respond and adapt to the external and internal stimuli. Many studies have focused on overall protein synthesis to understand how exercise regulates the muscular adaptation. However, despite the probability that each gene transcript may have its own unique translational characteristics and would be differentially regulated at translational level, little attention has been paid to how exercise affects translational regulation of individual genes at a genome-wide scale. Here, we conducted a genome-wide translational analysis using ribosome profiling to investigate the effect of a single bout of treadmill running (20 m/min for 60 min) on mouse gastrocnemius. Global translational profiles largely differed from those in transcription even at a basal resting condition as well as immediately after exercise. As for individual gene, Slc25a25 (Solute carrier family 25, member 25), localized in mitochondrial inner membrane and maintaining ATP homeostasis and endurance performance, showed significant up-regulation at translational level. However, multiple regression analysis suggests that Slc25a25 protein degradation may also have a role in mediating Slc25a25 protein abundance in the basal and early stages after acute endurance exercise.

## Introduction

The regulation and dynamics of protein synthesis and amino acid metabolism have been intensively investigated to understand the underlying mechanisms of muscle adaptation to exercise. The use of stable isotope tracers, for example, has advanced our understanding of the regulation of overall muscular metabolism of amino acids and protein synthesis [1–4]. It has been known that during acute endurance exercise global muscular protein synthesis decreases in human and rodents [5,6]. The fall in global protein synthesis can be largely mediated by activated AMPK (AMP-activated protein kinase) and decreased mTOR (mammalian target of rapamycin) signaling [7,8]. However, despite the intensive studies of global protein synthesis, some individual synthesis rates of proteins, and related signaling cascades [9,10], few studies have focused on the skeletal muscle protein synthesis or translational regulation of individual genes in a genome-wide scale. This is mainly due to the lack of methodology to investigate genome-wide translational dynamics [11].

In 2009, a pioneering study introduced a novel method, named ribosome profiling, to examine a genome-wide translational regulation using next generation sequencer [12]. In ribosome profiling [12,13], translating ribosomes are chemically stalled and mRNA regions that are not protected by ribosomes are enzymatically digested (i.e., mRNA regions that are not under translation at this point are removed). In contrast, mRNA regions that are protected by ribosomes, called ribosome-protected fragments (RPF) with the length of approximately 30 nucleotides (nt) in mammals, are extracted and deep-sequenced. As in standard high-throughput mRNA sequencing (mRNA-Seq) where highly expressed genes are indicated by more aligned sequenced reads, genes with more aligned RPF suggest more active translation. Therefore, using ribosome profiling, it is now possible to investigate the genome-wide translational regulation focusing on individual genes rather than overall picture of protein synthesis. Here, we carried out a genome-wide translational analysis to investigate the effect of a single bout of endurance exercise on mouse working muscle mainly focusing on highly expressed genes.

## Materials and Methods

### Accession number

The sequenced and processed files from this article have been deposited in the National Center for Biotechnology Information’s Gene Expression Omnibus under the accession number GSE69699.

### Animals and endurance exercise

Seven-week-old male C57BL/6J mice, purchased from Kiwa Laboratory Animals (Wakayama, Japan), were first acclimated to the lab environment for a week (temperature controlled at 23°C with 12 h light/12 h dark cycle and fed with normal chow) followed by acclimation to motorized treadmill (Natsume, Kyoto, Japan) for approximately 10 min. After another one week, mice were randomly allocated into five different groups: no exercise (n = 6), immediately after exercise (n = 6), post 1 h of exercise (n = 6), post 2 h of exercise (n = 6), and post 4 h of exercise (n = 6). The exercise group mice underwent treadmill running at 20 m/min for 1 h. At the indicated time points, translational inhibitor, 5 μl of 50 mg/ml cycloheximide (Sigma, St. Louis, MO) in PBS was injected into right gastrocnemius of all the mice (aged 9 to 10 weeks) under isoflurane anesthesia and the right gastrocnemius was immediately harvested, followed by brief wash in 100 μg/ml cycloheximide in PBS and flash-frozen in liquid nitrogen. The cycloheximide-treated samples were further processed for mRNA-Seq and ribosome profiling as described below. Left gastrocnemius was harvested for western blotting and real time PCR analyses. All the animal experiments were approved by the Institutional Animal Care and Use Committee at Waseda University (approval number: 2014-A085) in accordance with the guidelines of Ministry of Education, Culture, Sports, Science and Technology of Japan for animal experimentation at research institutes.

### Total RNA extraction for mRNA-Seq and ribosome profiling

Ribosome profiling protocols were modified from the original [12,13]. The right gastrocnemius was homogenized in 1.2 ml of ice-cold lysis buffer [20 mM Tris HCl pH7.4, 150 mM NaCl, 5mM MgCl_2_, 1 mM DTT, 100 μg/mL cycloheximide (Sigma, St. Louis, MO), TURBO DNase I 25 U/mL (Ambion, Life Technologies, Carlsbad, CA), and 1% Triton X-100 (Sigma)], followed by centrifugation at 15,000 g for 10 min at 4°C. Supernatant from the lysate was separated in two 600 μl aliquots; one for mRNA-Seq and the other for ribosome profiling. To avoid the statistical underestimation of exercise effect by the large variation of individual biological differences, we combined 3 different mouse muscles to prepare 1 biological replicate and created 2 totally independent biological replicates for each of the control and exercise groups. Therefore, three sets of 100 μl of the aliquot from 3 individual mice were combined together to create 1 biological replicate (i.e., 1 biological replicate is composed of 3 different mice both in mRNA-Seq and ribosome profiling).

### ribosomal RNA (rRNA) removal, 5′ phosphorylation, and 3′ dephosphorylation from RPF

RiboMinus (Invitrogen, Life Technologies, Carlsbad, CA) was used to remove rRNA from RPF samples. For both fragmented mRNA and rRNA-deleted RPF, 5′ phosphorylation and 3′ dephosphorylation were carried out. The 3′ ends were first dephosphorylated at 37°C for 60 min in reaction mixture [10 U/μl T4 polynucleotide kinase (New England Biolabs, Beverly, MA), T4 polynucleotide kinase buffer without ATP, and 20 U/μl SUPERase In (Ambion)], immediately followed by 5′ phosphorylation for 30 min in the presence of 1 mM ATP (New England Biolabs) and RNA participation.

### Sequence library preparation, sequencing, and alignment

We prepared sequence library using Ion Total RNA-Seq Kit v2 (Ion Torrent, Life Technologies, Carlsbad, CA) according to the manufacturer’s protocols except that reverse-transcribed cDNA was gel-purified to remove excess primer dimers. The region (approximately 63 nt) was excised and recovered by the same protocols as described above. Sequencing was carried out using Ion PGM Template OT2 200 Kit, Ion PGM sequencer, Ion PGM Sequencing 200 Kit v2, and Ion 318 Chip Kit v2, according to the manufacturer’s instructions (Ion Torrent). Base-calling and alignment were conducted using the built-in Ion Torrent software (V4.0.1) and the reads shorter than 24 nt were removed. Sequenced reads were mapped and aligned to coding DNA sequences extended to 25 nt upstream of translational start site of each gene, retrieved from GRCm38 using Biomart MartView (http://www.biomart.org/biomart/martview) [14]. As for reads with multiple hit, the best hit was aligned. All the figures and statistics below were created by the R software, if not otherwise specified.

### Reproducibility and validity of sequencing

To compare the reproducibility of two independent biological replicates, aligned read counts were normalized by total read count (RPM: Reads Per Million). For validation of ribosome profiling, triplet periodicity was confirmed. For each gene, 5´ end position of aligned reads was grouped into 0, -1, or +1 relative position to the main coding frame and the ratios of each frame were calculated. As for RPF density around the beginning of start codon, cumulated RPF counts throughout all the reference genes at each base position were divided by the average RPF counts (up to the first 140 nt).

To determine minimum threshold of read counts, read counts of one replicate were divided by total read counts composed of both replicates to calculate the fraction of one replicate. In a case of perfect reproducibility, the fraction would be 0.5. Based on average read counts, genes were binned and SD of the fraction was computed for each bin. The SD was predicted using binominal partitioning of the read counts. The variation in the binominal partitioning, derived from counting errors, should be smaller than the variation in the biological replicates to clearly take into account of the biological variations in the statistical analysis of differentially expressed genes. Therefore, a minimum read count, 125 RPM, was selected because variation between biological replicates becomes stably larger than that predicted from counting statistics approximately from 125 RPM.

### Sequenced data analysis

Focusing on 1011 genes (>125 RPM), principal component analysis was carried out. We plotted PC1 and PC3, well representing the differences between the profiles of transcription/translation and no exercise/exercise, respectively. To detect differentially regulated genes at transcriptional and translational levels, edgeR was used with TMM normalization [15,16]. For the differentially regulated genes, pathway enrichment analysis was conducted using InnateDB [17] with hypergeometric algorithm and Benjamini Hochberg *P*-value correction where pathways in KEGG [18] were only focused. As for the known TOP motif genes, we focused only on highly expressed genes (> 125 RPM). Translational efficiency was obtained by dividing normalized RPF counts by normalized mRNA-Seq counts. Kolmogorov-Smirnov test was used for statistics.

### Real time qPCR and western blotting

Total RNA was extracted using TRIzol (Invitrogen), followed by cDNA synthesis using High Capacity cDNA Reverse Transcription Kit (Applied Biosystems, Life Technologies, Carlsbad, CA) according to manufacturer’s instruction. Gene expressions were determined using SYBR Green (Applied Biosystems) with primers of Slc25a25, Mmp2 (Matrix metalloproteinase-2), Nrf2 (Nuclear Factor (erythroid-derived 2)-like 2), Atrogin1, MuRF1 (Muscle RING-Finger Protein-1), Pmpca (Peptidase mitochondrial processing alpha), Yme1l1 (YME1-like 1 ATPase), and Immp2l (IMP2 inner mitochondrial membrane peptidase-like) (Takara Bio, Kyoto, Japan). For internal control, Actin beta (Takara Bio) was used. Protein was extracted using PARIS kit (Ambion) supplemented with protease inhibitor cocktails (Thermo Fisher Scientific, Carlsbad, CA) according to manufacturer’s instruction. For western blotting, 5 μg of total protein was loaded into 4–12% Bis-Tris precast polyacrylamide gel (Thermo Fisher Scientific), followed by electrophoresis and transferring to PVDM membrane using iBlot 2 system (Thermo Fisher Scientific). Anti-SLC25A25 (GeneTex, Irvine, CA) and anti-GAPDH (Cell Signaling, Danvers, MA) for internal control were used and ECL (GE Healthcare, Pittsburgh, PA) was used for detection. The experiments were repeated to confirm the reproducibility. As for statistics, one-way ANOVA with Tukey was used. Based on the results of real time qPCR and western blotting, Pearson correlations were calculated between Slc25a25 protein abundance and other gene expressions in each group. The correlation patterns were visualized by Cytoscape [19]. We then carried out multiple linear regression analysis using the expression levels in the groups: no exercise, immediately after exercise, and post 1 h of exercise, in which all the genes were initially included to validate the model and then the least significant gene was repeatedly removed at each validation (i.e., Slc25a25 mRNA, Atrogin1, MuRF1, and Yme1l1 were removed).

## Results

**Fig 1:**
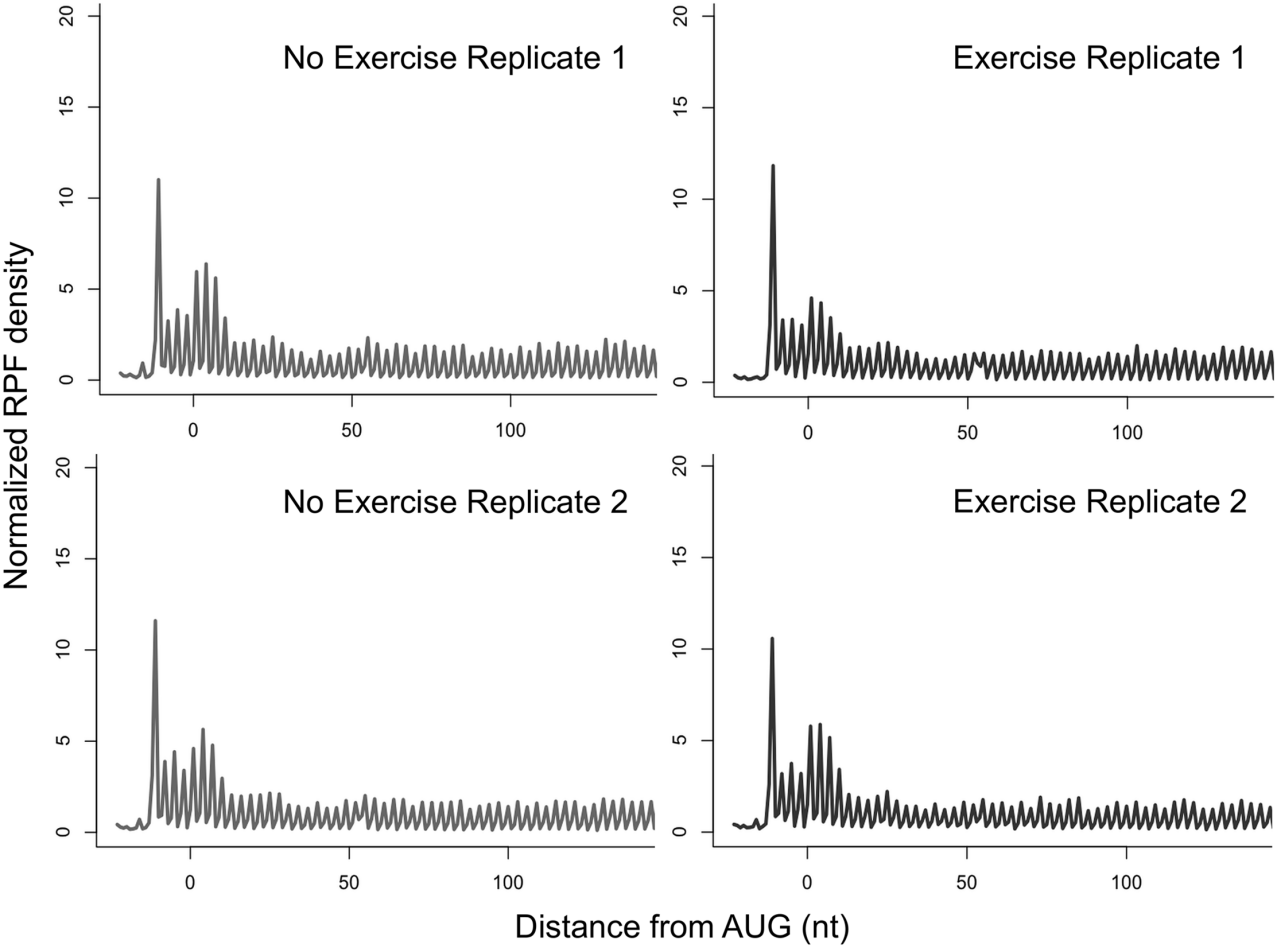
Validity of ribosome profiling data. Metagene analysis of normalized RPF density at the corresponding base positions relative to the start codon was shown for each replicate.

We conducted a genome-wide analysis using mRNA-Seq and ribosome profiling to investigate the transcriptional and translational regulation in mouse gastrocnemius during a single bout of endurance exercise. Two fully independent biological replicates showed highly reproducible outcomes (S1 Fig). Importantly, successful ribosome profiling data shows unique characteristics of the aligned reads, exemplified by triplet periodicity and a sharp RPF peak at the translational start sites [12,20]. This triplet periodicity comes from the ribosomal kinetics during translation. Because translating ribosomes move by 3 nt by 3 nt on mRNA, read counts of the 5´ end of aligned RPF results in uneven distribution, in which the strongest peak at a coding frame indicates the main coding frame [12,20]. This striking feature was only present in ribosome profiling but not in mRNA-Seq (Fig 1, S2 Fig). Moreover, we also observed a sharp RPF peak in all the RPF replicates exactly 4 codons upstream of the start codon (Fig 1), which perfectly agrees with the previous analyses [20,21]. The strong RPF peak reflects relatively longer resident time of ribosomes at the start codon. It is known that when harvesting cells without flash-freezing or no translational inhibitor, the RPF peak near start codon disappears due to the failure of successful stall of ribosomes [12]. Given the observed unique features in ribosome profiling, the results support that translational profiles of skeletal muscle with or without endurance exercise were successfully captured in the current study.

**Fig 2:**
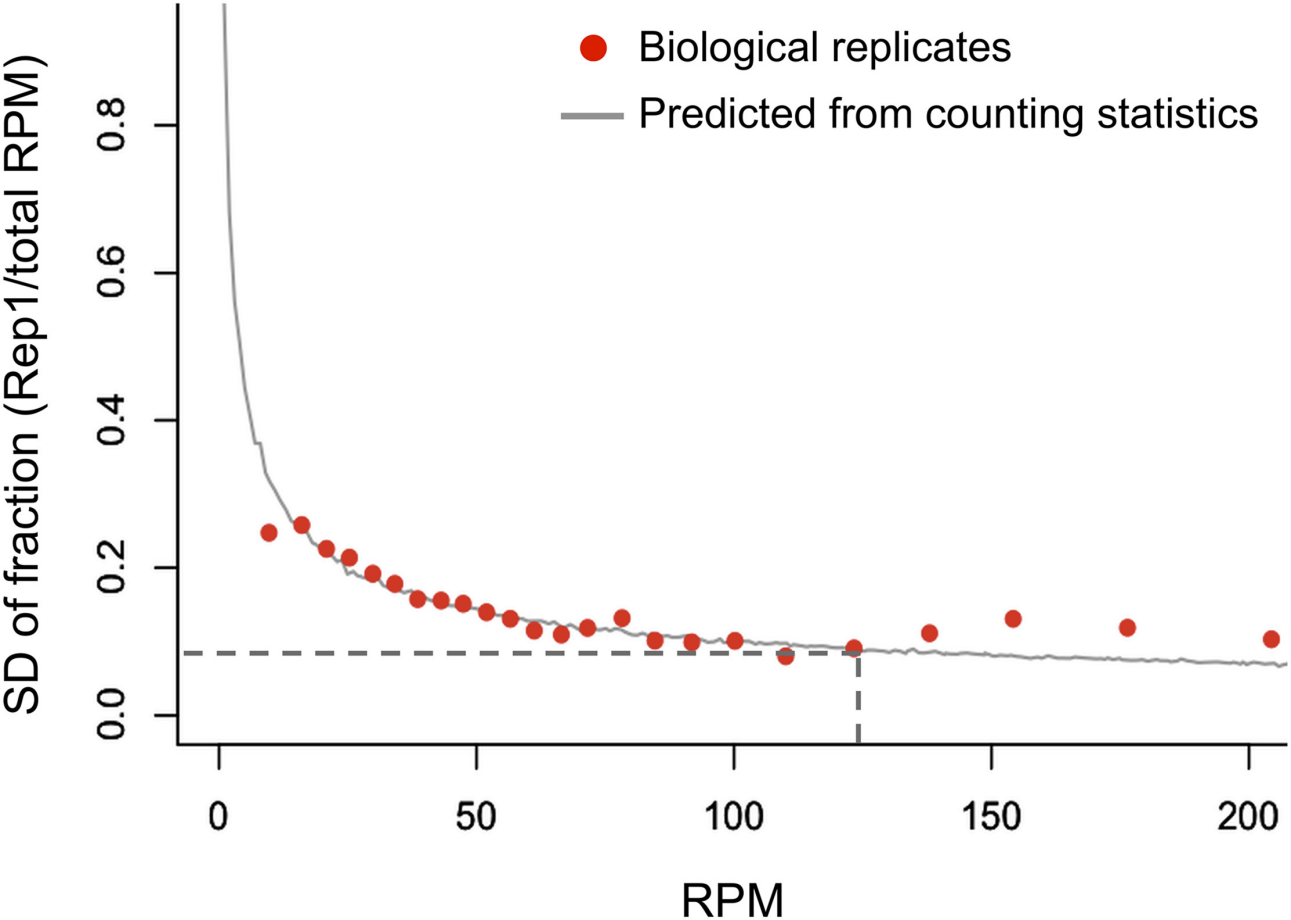
Determination of minimum read counts for downstream analyses. For reliable downstream measurements, minimum read counts were determined using the independent biological replicates of ribosome profiling (immediately after exercise). For each gene, the fraction of reads (read counts of one replicate divided by total read counts derived from both replicates) was calculated. Based on average read counts, genes were binned and standard deviation (SD) of the fraction was computed for each bin. The SD for counting statistics between the replicates was predicted using binominal partitioning of the read counts. The minimum read count (125 RPM) is indicated by a dashed line from where error between biological replicates becomes stably larger than error from counting statistics. RPM: Reads Per Million.

We then determined minimum expression threshold for reliable quantification of transcription and translation of genes. To this end, we measured the reproducibility of the independent biological replicates in ribosome profiling and compared variation in the inter-replicates with that from counting statistics [12,22] (Fig 2). For the genes with less aligned reads, binominal partitioning predominated the variation in the biological inter-replicates (i.e., variation predicted from counting statistics was similar to that in the inter-replicates). However, when more reads are available, approximately more than 125 RPM, the effect of binomial partitioning was attenuated and other sources of variation (e.g., individual differences) became larger (i.e., variation in the inter-replicates was larger than that from counting statistics). Therefore, we selected the genes with more than 125 RPM for downstream analyses.

**Fig 3:**
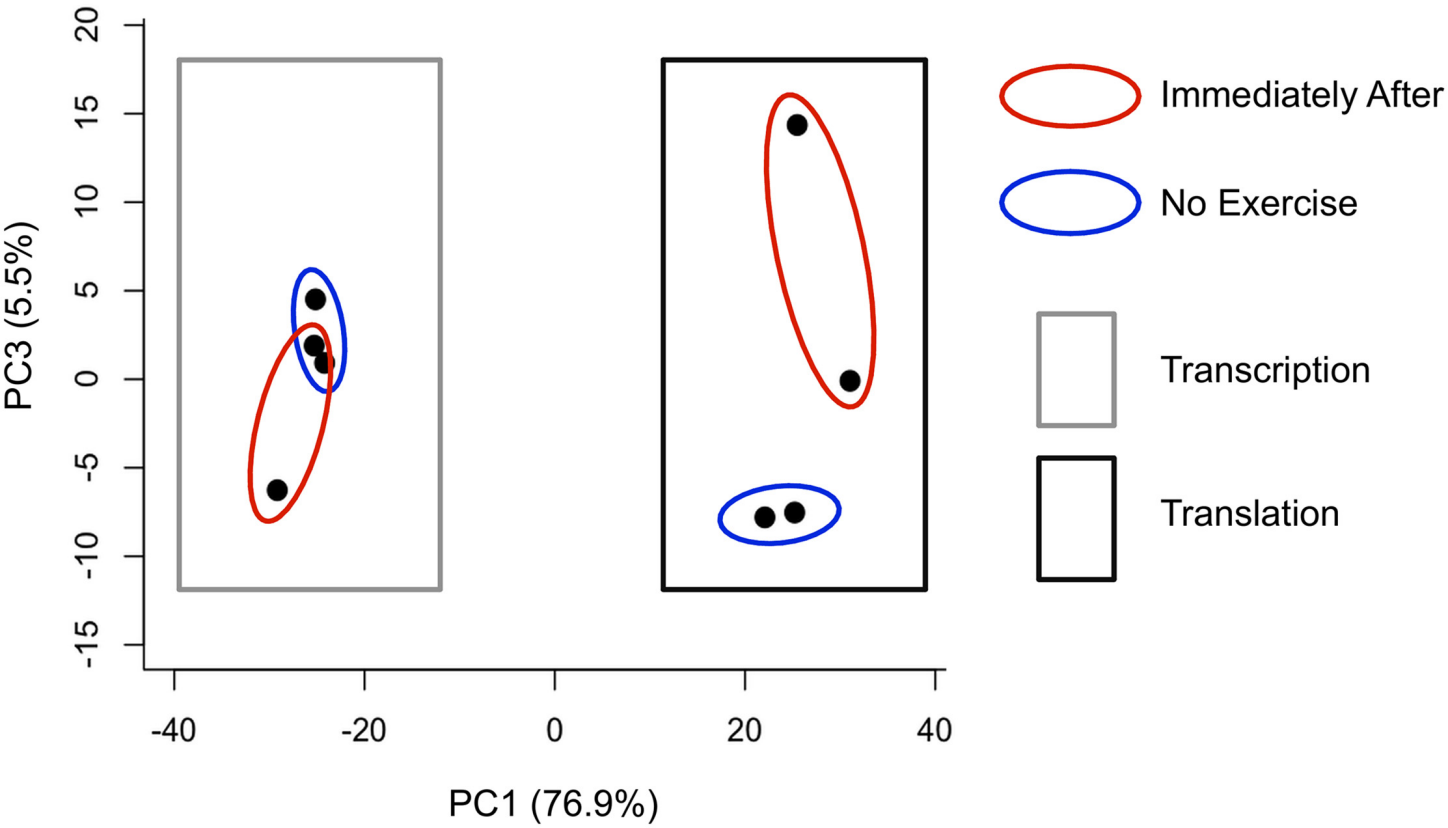
Principal component analysis of mRNA-Seq and ribosome profiling. Principal component analysis was conducted for each replicate targeting genes with more than 125 RPM (n = 1011). The replicates belonging to the same condition were circle together. The replicate derived from mRNA-Seq or ribosome profiling was surrounded by a rectangle colored in grey or black, respectively.

To investigate the global features of transcriptional and translational profiles, principal component analysis was carried out (Fig 3). Intriguingly, there were notable distinctions between transcription and translation as well as with and without exercise. The translational profiles were clearly different from those in transcription independent of exercise stimuli. Exercise-induced changes in translational profiles were larger than those in transcription. These results suggest that majority of translation and transcription can be regulated separately even at a basal resting condition and that exercise can induce larger changes in translation than in transcription immediately after a single bout of endurance exercise.

To identify differentially expressed genes at transcriptional and translational levels, we compared the expression with and without exercise (S3 Fig). At transcriptional and translational levels, 26 and 37 genes were differentially expressed (*P* < 0.01), respectively (S1 Table). Using those genes, pathway enrichment analysis was conducted and showed that a pathway related to amino acid metabolism was commonly enriched both in transcription and translation (S2 and S3 Tables). Other pathways, associated with innate immunity, generic transcription, ion channel transport, phagosome, and glucose metabolism, were enriched only in the differentially regulated translation genes (S2 and S3 Tables).

**Fig 4:**
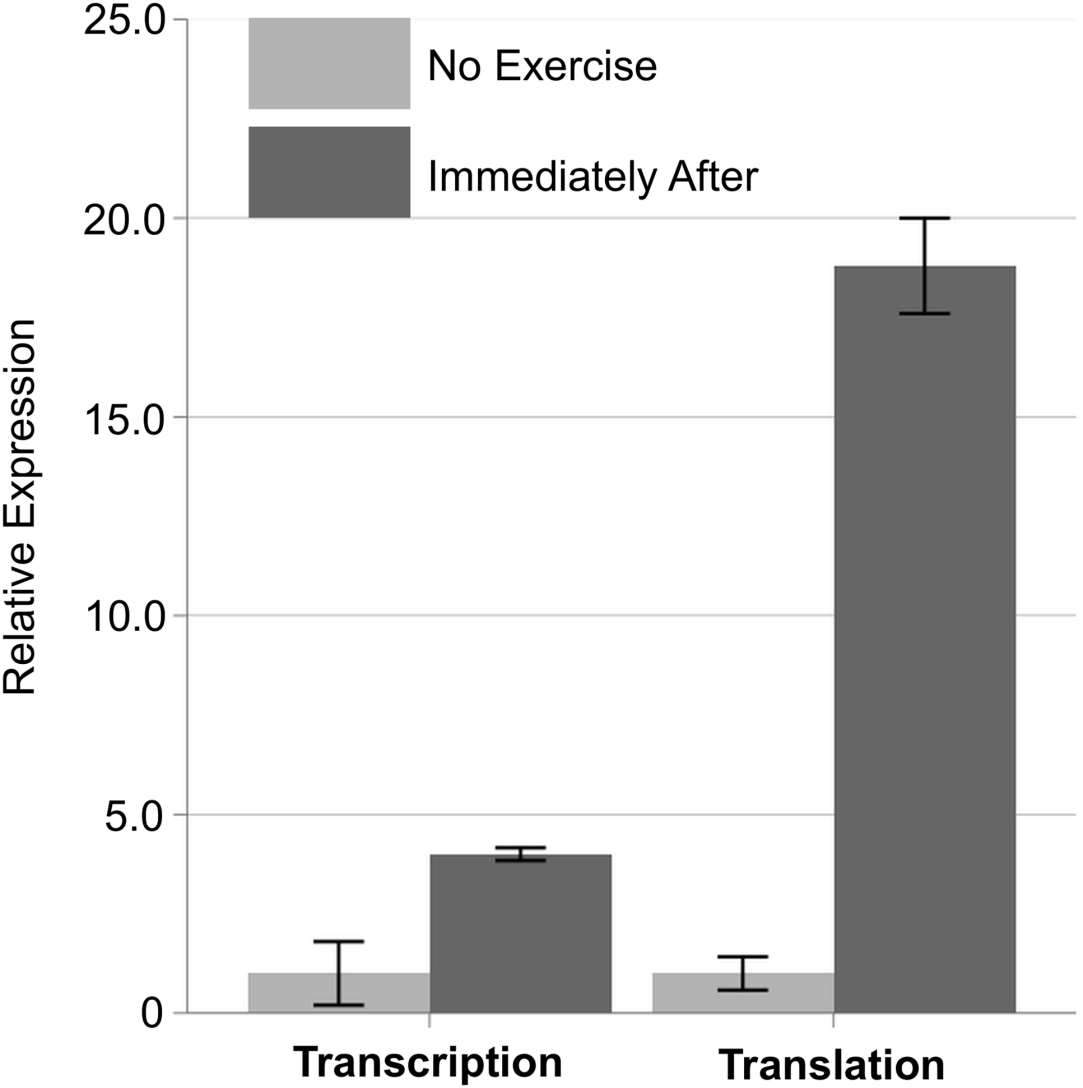
Quantification of transcription and translation levels of Slc25a25. The relative abundance of Slc25a25 transcription and translation, measured by mRNA-Seq and ribosome profiling, respectively, were compared with and without exercise using ribosome profiling. Mean ± SD.

Closely focusing on the differentially regulated genes, we selected Slc25a25 for further analyses. Slc25a25 is a mitochondrial protein residing on the mitochondrial inner membrane and having a critical role in maintaining ATP homeostasis [23,24]. The reasons why we selected Slc25a25 are: 1) Slc25a25 is one of the most differentially regulated genes at translational level, 2) Slc25a25 exhibited a significantly different regulation between transcriptional and translational levels, 3) knockout of Slc25a25 reduces the endurance performance during treadmill running [25], and 4) to our knowledge, the effect of exercise, including both resistance and endurance, on Slc25a25 dynamics has never been investigated. At transcription level, although mRNA-Seq showed a moderate expression change (approximately 4 fold increase), ribosome profiling showed much greater increase (approximately 18 fold) in translation level (Fig 4). These results suggest that immediately after a single bout of endurance exercise, Slc25a25 expression can be regulated predominantly by translation level rather than transcription level.

**Fig 5:**
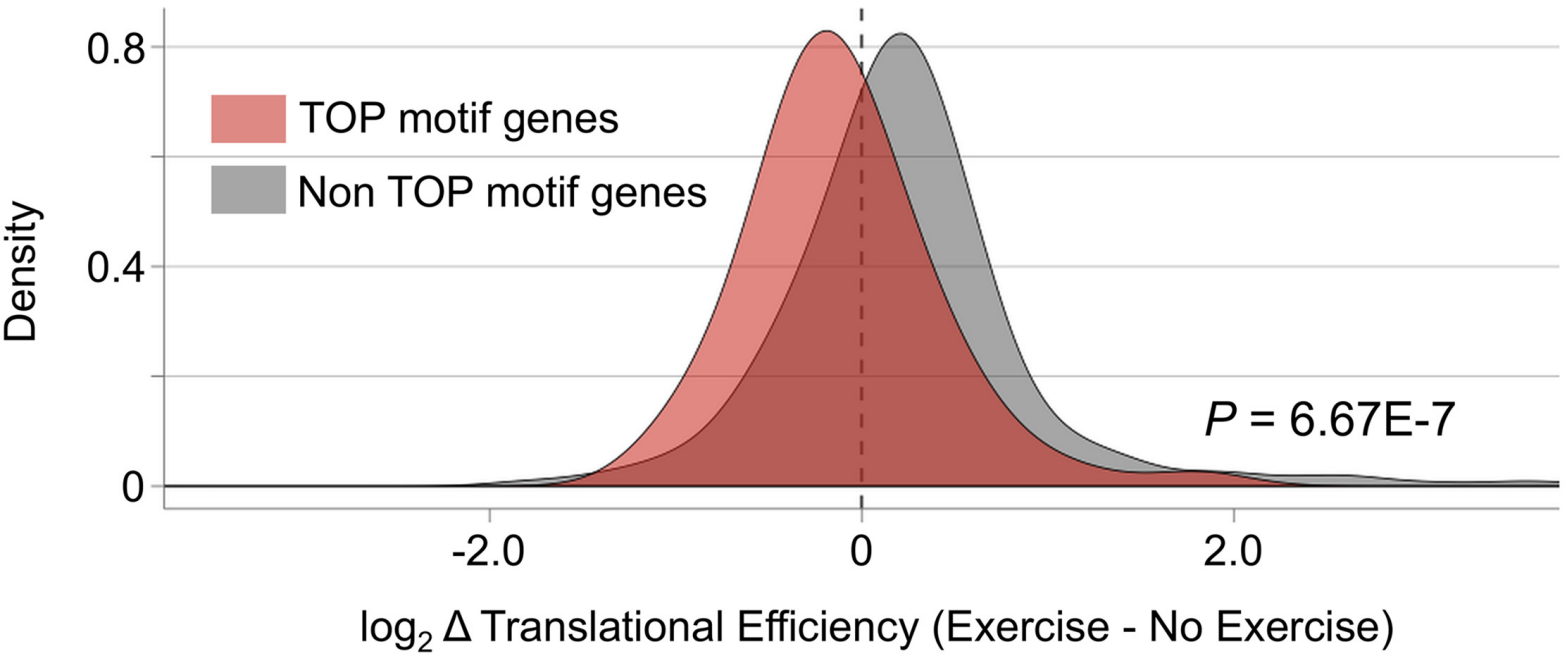
Exercise-induced change in translational efficiency of TOP and non TOP motif genes. Log-transformed translational efficiencies (RPF/mRNA-Seq) and the ratios (exercise/no exercise) were calculated for known TOP motif genes (n = 59) and the other non TOP motif genes (n = 953). Kolmogorov-Smirnov test was used for statistics.

mTOR signaling is a widely recognized translational regulator and activation of the cascade enhances translational initiation. However, it has been known that acute endurance exercise can decrease global protein synthesis [5,6] and mTOR signaling can be associated with the reduction [7,8]. Given this, it is unlikely that the currently observed translational up-regulation of Slc25a25 protein is mediated by mTOR signaling. To support this, we investigated the effect of mTOR on translational regulation by focusing on TOP motif mRNA, known as pyrimidine rich sequence immediately downstream of 5′ cap [26] and functionally enriched in ribosome/ translation (S4 Table). These TOP and TOP-like mRNA are highly prone to mTOR suppression [26]. By analyzing the translational changes in these genes, we can assume whether mTOR signaling is suppressed and has negative impact on translation under a condition. As exprected, acute endurance exercise decreased translational efficiency of the known TOP genes compared with that in the other genes (Fig 5), which is consistent to previous studies reporting the reduced overall protein synthesis during endurance exercise [7,27]. Taken together with the results and that Slc25a25 mRNA is neither TOP or TOP-like mRNA, the enhanced translation of Slc25a25 could be regulated by unknown factors rather than mTOR signaling.

**Fig 6:**
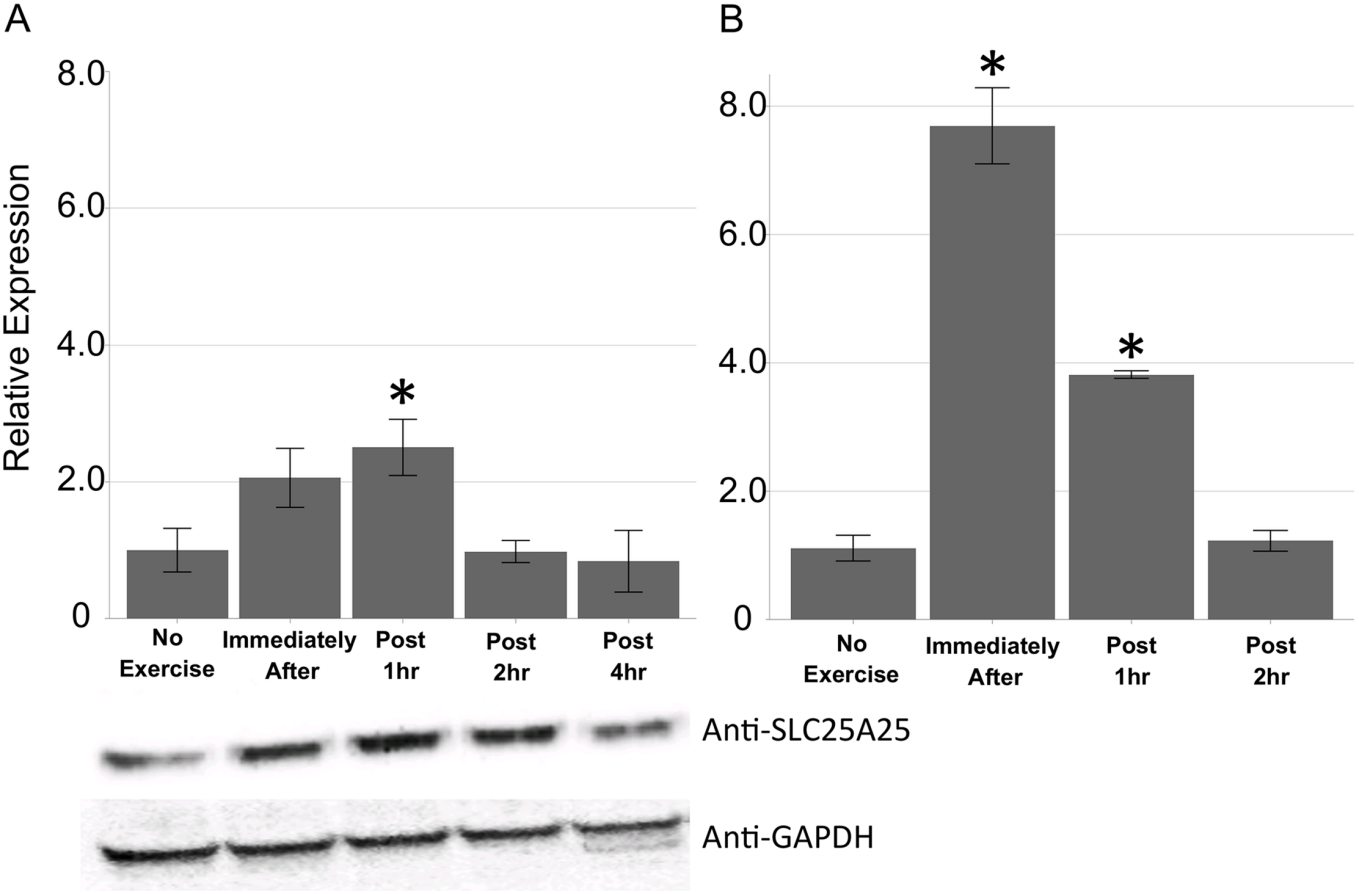
Quantification of transcription and protein levels of Slc25a25. (A) Slc25a25 protein abundance was analyzed by western blotting (n = 6). Mean ± SE. One-way ANOVA with Tukey (* *P* < 0.01). (B) Slc25a25 mRNA level was measured by real time PCR (n = 6). Mean ± SE. One-way ANOVA with Tukey (* *P* < 0.01).

Because Slc25a25 translational up-regulation exceeded the increase in transcription, Slc25a25 protein abundance may be much greater during/after acute endurance exercise than that predicted from the transcription level. To confirm that Slc25a25 protein abundance is regulated mainly by translational change rather than transcriptional change, Slc25a25 protein level was measured by western blotting and the transcript level was validated by real time qPCR (Fig 6A and 6B). Surprisingly, Slc25a25 protein abundance increased by much smaller extent compared with translation or even transcription. Slc25a25 transcript level showed approximately 7 fold increase immediately after exercise, which is comparable to the fold increase observed in mRNA-Seq. However, Slc25a25 protein expression increased by only approximately 2 fold immediately after and post 1 h of exercise even though its translation level rose by approximately 18 fold (Fig 4). These results imply the effect of post-translational regulation, such as protein degradation.

**Fig 7:**
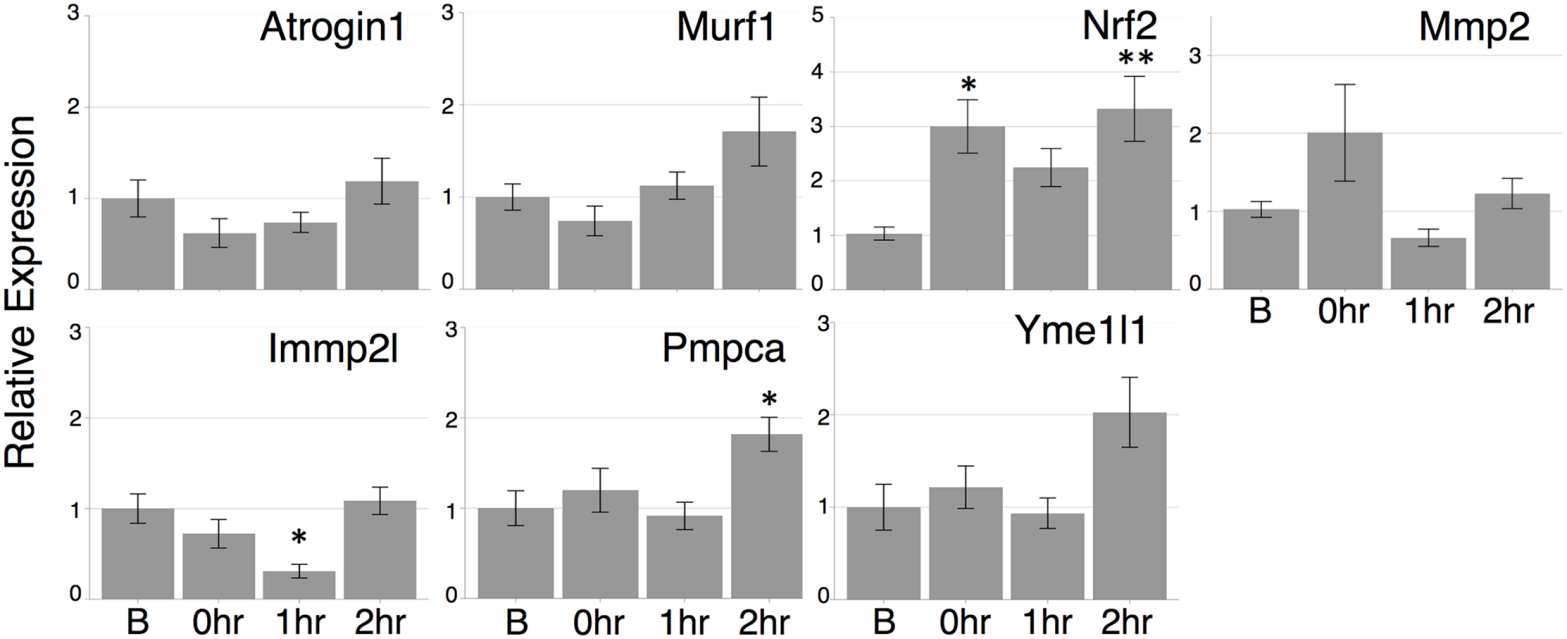
Measurements of the expression levels of proteolysis related genes. Genes related to proteolysis were measured by real time qPCR (n = 6). Mean ± SE. One-way ANOVA with Tukey (** *P* < 0.01, * *P* < 0.05).

To account for the striking discrepancy between the translation and protein level, we next investigated key players of post-translational regulation, especially protein degradation. Atrogin1 and MuRF1, well-recognized regulators in muscle protein degradation, were examined. Mmp2 and Nrf2 also have been known to have critical roles in cation-mediated proteolysis and proteasome-mediated protein degradation, respectively [28,29]. Both genes are induced by exercise stimuli [6,30,31]. Because Slc25a25 protein is a mitochondrial protein, genes regulating proteolysis in mitochondria, Yme1l1, Immp2l, and Pmpca, were also investigated. Yme1l1 is located in mitochondrial inner membrane and functions as ATP-dependent protease. Immp2l and Pmpca are both responsible for presequence cleavage, though Immp2l is present in inner membrane and Pmpca is found in mitochondrial matrix [32]. We observed that a single bout of exercise immediately increased expression of Nrf2 (approximately 3 fold increase) at statistically significant level and Mmp2 (approximately 2 fold increase) not statistically significant, though Atrogin1 and MuRF1 showed decreased tendencies but not at statistically significant levels (Fig 7). Although the mitochondrial genes, Immp2l, significantly decreased its expression at 1 h after exercise, the expressions of Pmpca and Yme1l1 increased at 2 h post exercise (Fig 7).

**Fig 8:**
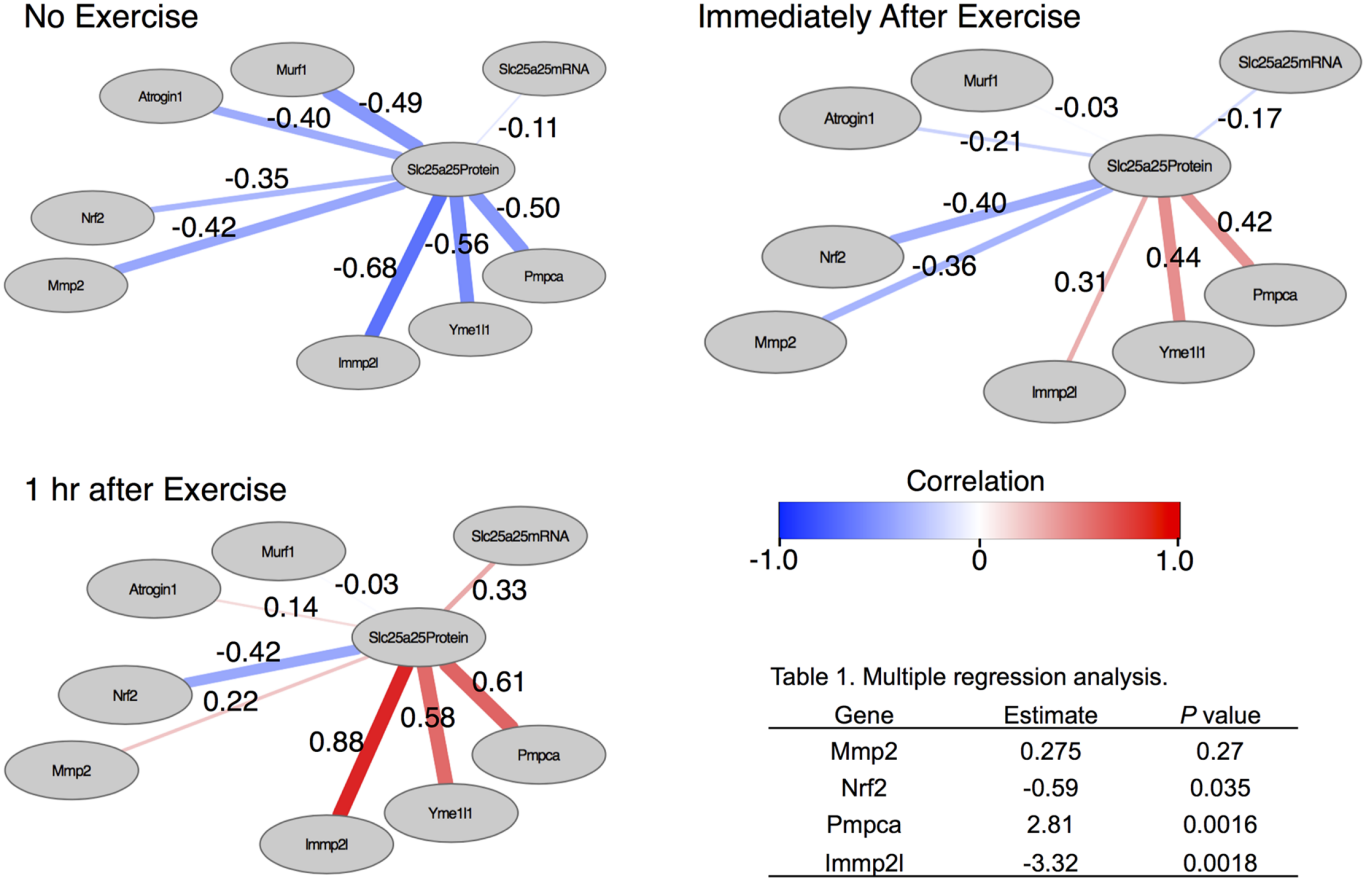
Correlation and multiple regression analyses. Association strength was measured among the protein abundance of Slc25a25 and gene expressions of Slc25a25, Mmp2, Nrf2, Atrogin1, MuRF1, Immp2l, Yme1l1, and Pmpca using simple correlation analysis and multiple linear regression analysis (Table 1). In the table of the regression analysis, an estimated coefficient of each gene and a corresponding *P* value are shown.

To further investigate the strength of the association among Mmp2, Nrf2, Slc25a25 mRNA, Atrogin1, MuRF1, Immp2l, Pmpca, Yme1l1 and Slc25a25 protein, we conducted a correlation analysis and multiple regression analysis (Fig 8, Table 1). Interestingly, contribution of Slc25a25 mRNA to Slc25a25 protein abundance was weak throughout the 3 different time points (Fig 8). On the other hand, multiple regression analysis suggested that Nrf2 and Immp2l had negative effects on Slc25a25 protein abundance, though Pmpca was positively associated with Slc25a25 protein abundance (Table 1)

**Table 1:** Multiple regression analysis.

Gene	Estimate	*P* value
Mmp2	0.275	0.27
Nrf2	-0.59	0.035
Pmpca	2.81	0.0016
Immp2l	-3.32	0.0018

## Discussion

We conducted a genome-wide translational analysis using ribosome profiling to investigate the effect of acute endurance exercise on mouse gastrocnemius. However, there are some limitations in the current studies. One is that we could measure the global translational profiles only immediately after the exercise. Moreover, due to relatively smaller total read counts we obtained, we could analyze only highly translated or/and expressed genes. Therefore, we could miss other intriguing regulations that are outside of the current time-courses or below the detection threshold in this study. Another limitation is that ribosome profiling cannot discriminate the translational profile of many mRNAs with monosome (single ribosome on mRNA) from that of single mRNA with polysome (multiple ribosomes on mRNA). Translation of mRNA can be upregulated by either increasing the numbers of translating ribosomes on mRNA or increasing the numbers of mRNAs loaded with a ribosome (or combination of both). However, ribosome profiling cannot distinguish such differences. Therefore, we cannot discriminate whether the currently observed translational upregulation/downregulation were derived from increasing/decreasing ribosomes on mRNA or mRNAs with ribosomes.

Overall, our analysis revealed a remarkable distinction between transcriptional and translational profiles even under a basal resting condition. At translational levels, as other studies have reported that acute endurance exercise could decrease mTOR signaling and therefore reduce protein synthesis [7,8], we consistently observed that TOP-motif genes, prone to the inhibition of mTOR signaling, reduced the translational efficiency. Acute endurance exercise induced dynamic changes both in transcription and translation but with larger extent in translational profiles, exemplified by Slc25a25. Further analyses suggest that transcriptional regulation of Slc25a25 little contributes to Slc25a25 protein abundance but that both translational up-regulation and protein degradation could be the key to maintain Slc25a25 protein abundance in the conditions without and early stages after acute endurance exercise. Although the mechanism for the upregulated Slc25a25 translation is unclear, given the decreased translational efficiencies in the TOP-motif genes, mTOR signaling cascade seems not to be involved in upregulating Slc25a25 translation immediately after acute endurance exercise.

Highly enhanced Slc25a25 translation might be traded off by Nrf2-mediated enhanced proteolysis. Our data showed that acute endurance exercise enhanced Slc25a25 translation by approximately 18 fold. However, the corresponding protein abundance increased only by approximately 2 to 2.5 fold. This huge discrepancy might be explained by promoted proteolysis activity mediated by Nrf2. Nrf2 promotes the gene expression of the components of proteasome complex, including 20S, 19S, and 11S [29]. Increasing cytosolic Nrf2 level is reported to be necessary to increase proteasome activity [33]. In the current study, Nrf2 expression was significantly increased by acute endurance running. As the regression analysis suggested, upregulated Nrf2 might have a role in cancelling the elevated translational levels of Slc25a25.

Our data suggested that Pmpca and Immp2l could have positive and negative impact on Slc25a25 protein abundance, respectively. Both Pmpca and Immp2l are mitochondrial proteins responsible for presequence cleavage, though their functions are different. When nuclear-encoded mitochondrial proteins are imported into mitochondria, Pmpca cleaves them to be properly folded and functionally matured [34,35]. Given that nuclear-encoded Slc25a25 protein has to be processed to properly localize in inner membrane without aggregating or improperly degraded, Pmpca could be responsible for Slc25a25 protein maturation without degradation, supporting the positive effect of Pmpca on Slc25a25 protein abundance. On the other hand, Immp2l, located in inner membrane, has specific target substrates and the defect in Immp2l can lead to increased mitochondrial ATP levels [36]. Considering that the loss of Slc25a25 results in decreased mitochondrial ATP levels [25] (i.e., the phenotype is negatively correlated with that of Immp2l deficiency), as suggested in the current analysis, Immpl2 might have a negative influence on Slc25a25 protein abundance.

To our knowledge, this is the first preliminary study to investigate the effect of exercise on genome-wide translation of skeletal muscle and to focus on the translational regulation of individual genes. Translational profiles significantly differed from those of transcription even under the basal state. The results suggest that acute endurance exercise can enhance the translation of Slc25a25. However, the regression analysis also suggests that Slc25a25 protein degradation may also have a role in maintaining Slc25a25 protein abundance especially early after acute endurance exercise. Because of decreasing running cost of next generation sequencer, it is now easier to apply genome-wide analysis, which enables us to examine translational regulation of individual genes. Some of them can be largely regulated by the intensively characterized mTOR signaling cascades. However, translation of other genes can be mediated by many other known and unknown translational regulators, such as mRNA secondary structure and associated proteins, microRNA, translation speed, or translational stall. We believe that more focus on individual translational regulation would shed novel light on underlying mechanisms of muscular adaptation to exercise.

## Supporting Information

S1 Fig Reproducibility of mRNA-Seq and ribosome profiling. Pearson correlations of biological replicates (log_2_ scale aligned reads) are shown for the skeletal muscle without exercise (No Exercise) or immediately after a single bout of exercise (Exercise). RPM: Reads Per Million.
(PDF)

S2 Fig Validity of ribosome profiling data. Triplet periodicity of RPF for the biological replicates is shown for the skeletal muscle without exercise (No Exercise) or immediately after a single bout of exercise (Exercise). The ratio of the read counts corresponding to one of the coding frames were calculated. The main coding frame is represented by “0”. Mean ± SD.
(PDF)

S3 Fig Transcriptional or translational changes with or without exercise. Log-transformed expression ratio was compared using MA plot targeting genes with more than 125 RPM (n = 1011). Differentially expressed genes (*P* < 0.01) were determined by edgeR and colored in red.
(PDF)

S1 Table Top 100 genes and the corresponding expression (RPM) analyzed in differential expression analysis are listed. (XLSX)

S2 Table Pathway enrichment in the differentially regulated genes at transcription level. (XLSX)

S3 Table Pathway enrichment in the differentially regulated genes at translation level. (XLSX)

S4 Table Gene ontology enrichment in the known TOP motif genes that expressed in the current study (> 125 RPM). (XLSX)
