# Supplementary figures and images for: Genome-Wide Analysis of Acute Endurance Exercise-Induced Translational Regulation in Mouse Skeletal Muscle
Source: PLoS One. 2016 Feb 4;11(2):e0148311. doi: 10.1371/journal.pone.0148311 (PMC4742069; doi:10.1371/journal.pone.0148311)

Biological replicate 1 ( $\log_2\text{RPM}$ )

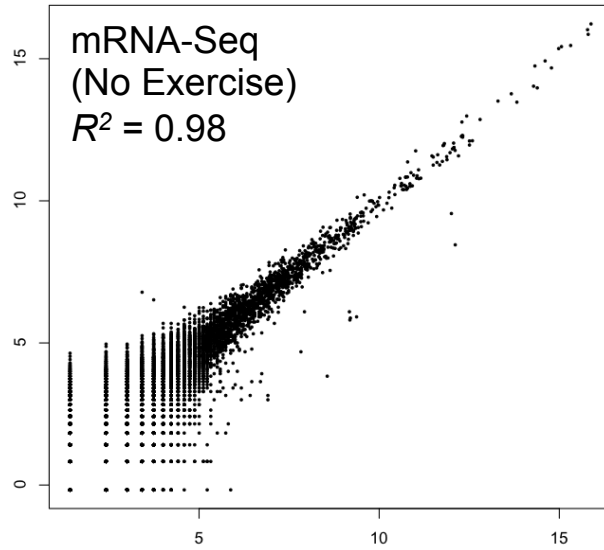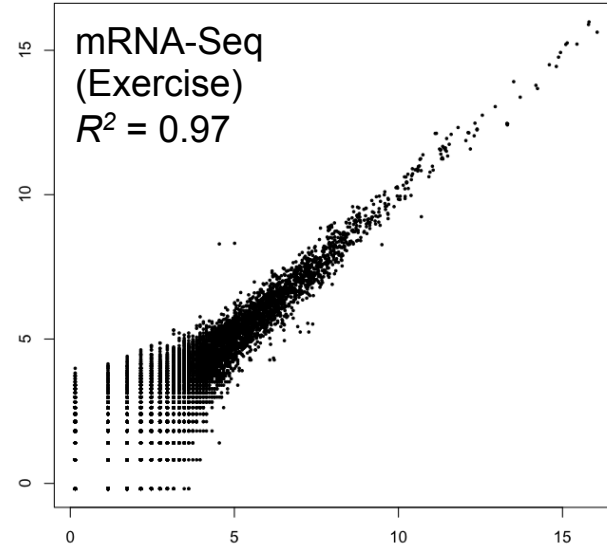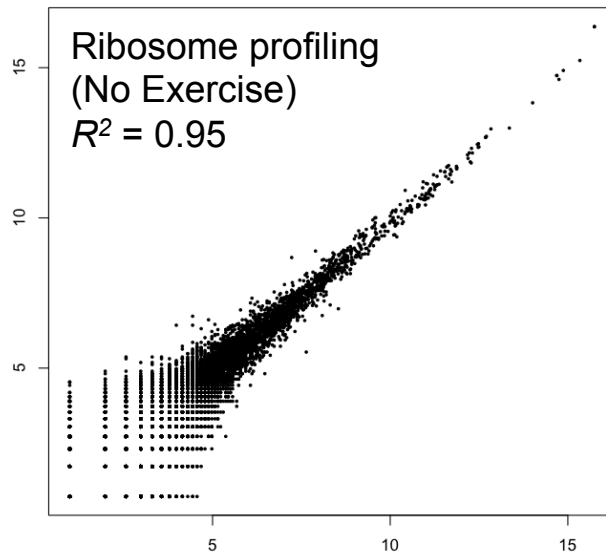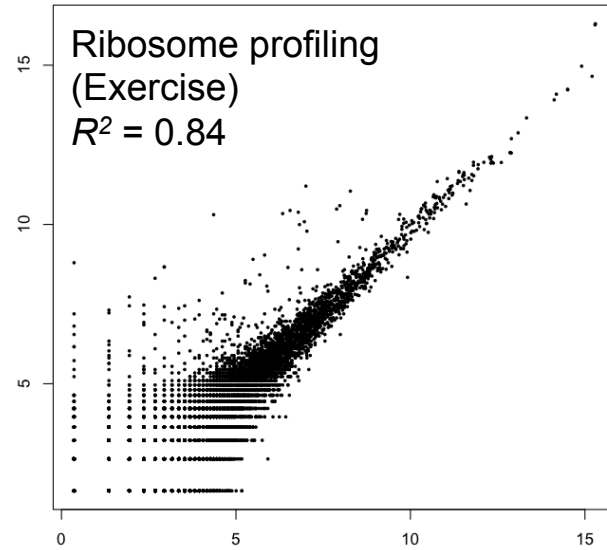

Biological replicate 2 ( $\log_2\text{RPM}$ )

Supplement: S1 Fig — Pearson correlations of biological replicates (log2 scale aligned reads) are shown for the skeletal muscle without exercise (No Exercise) or immediately after a single bout of exercise (Exercise). RPM: Reads Per Million. (PDF) [file pone.0148311.s001.pdf]

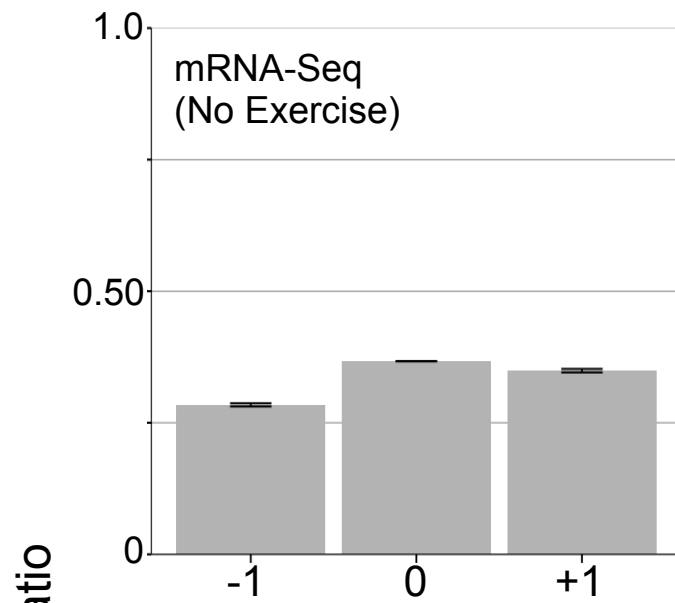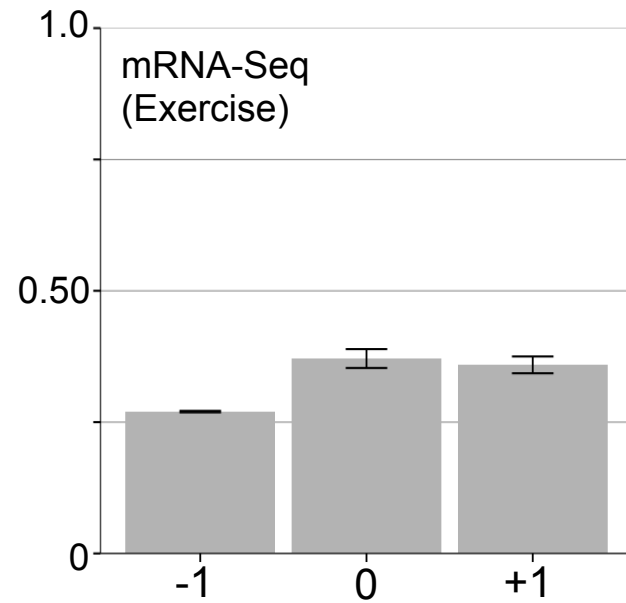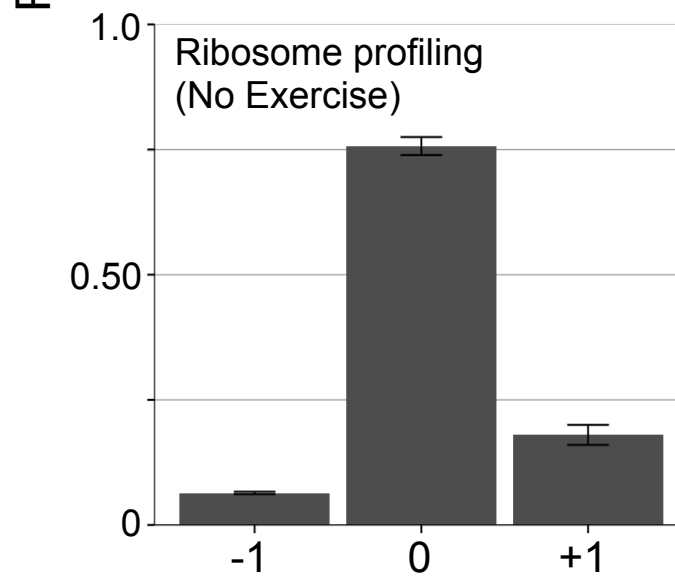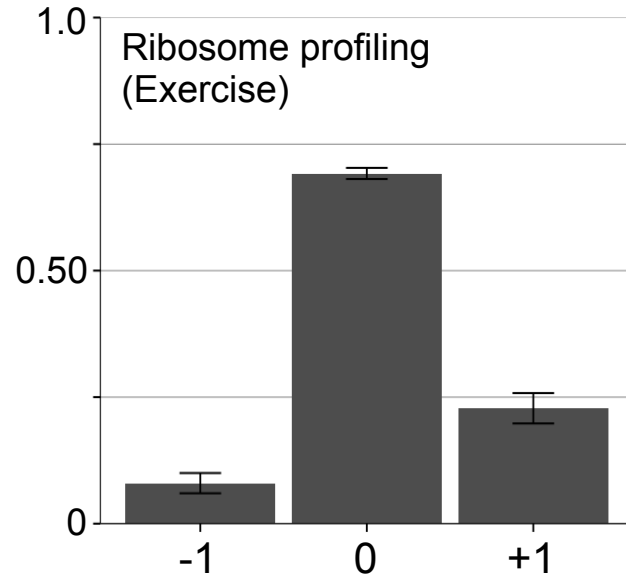

Coding frame (relative to main coding frame)

Supplement: S2 Fig — Triplet periodicity of RPF for the biological replicates is shown for the skeletal muscle without exercise (No Exercise) or immediately after a single bout of exercise (Exercise). The ratio of the read counts corresponding to one of the coding frames were calculated. The main coding frame is represented by “0”. Mean ± SD. (PDF) [file pone.0148311.s002.pdf]

## Transcription

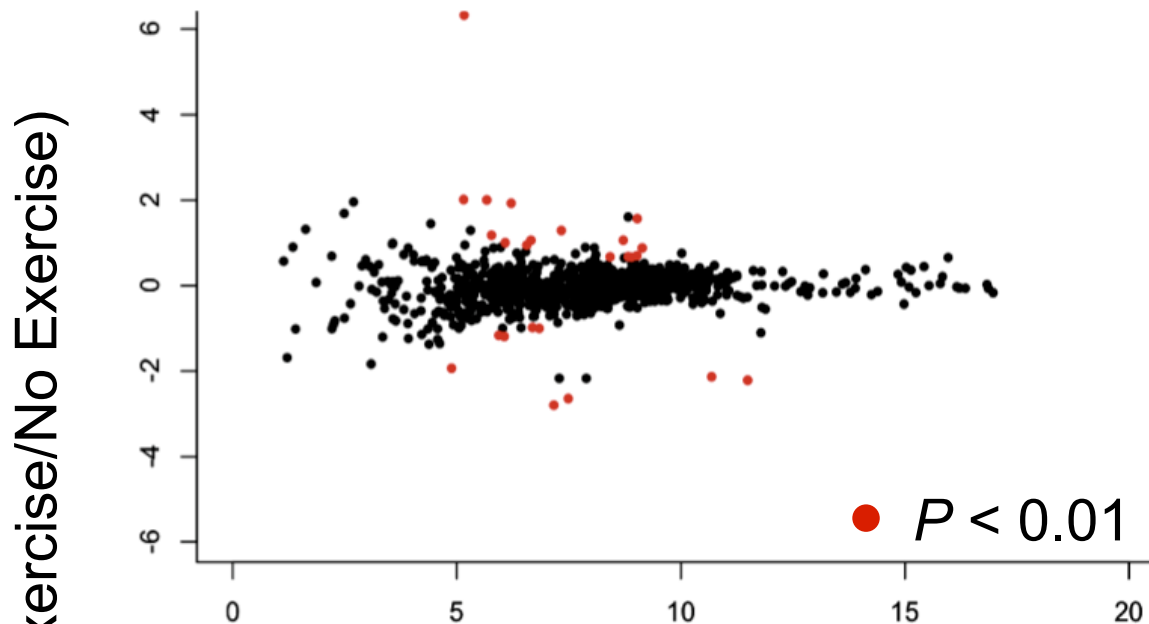

## Translation

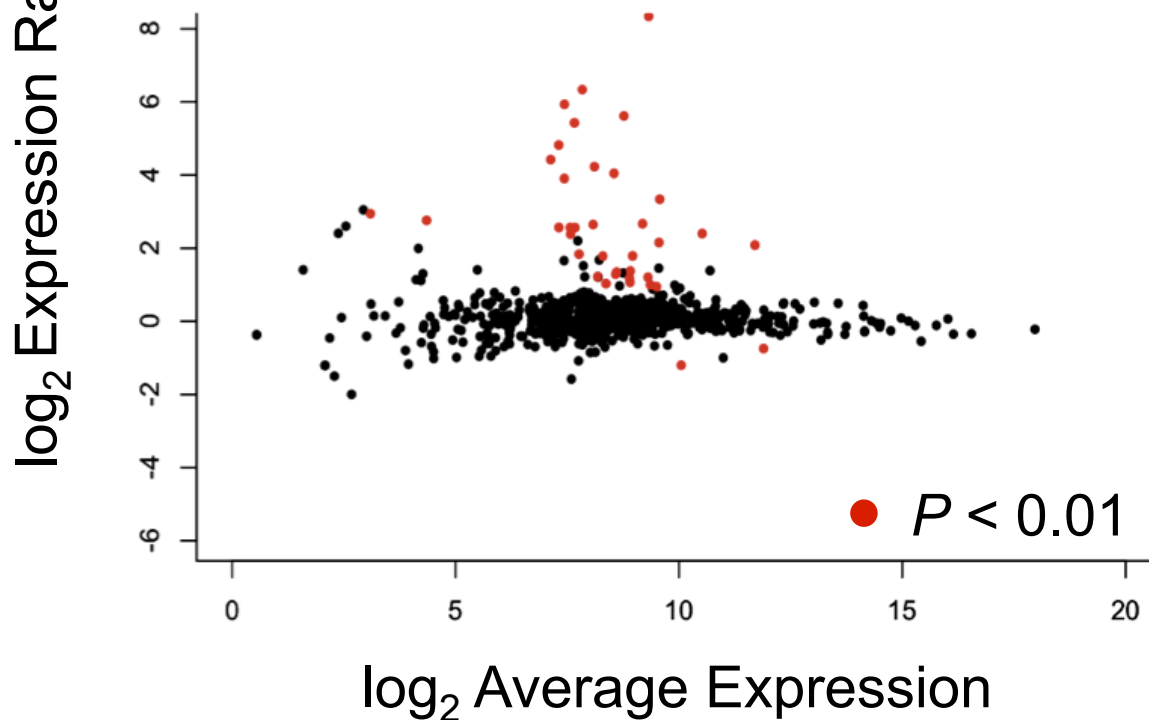

Supplement: S3 Fig — Log-transformed expression ratio was compared using MA plot targeting genes with more than 125 RPM (n = 1011). Differentially expressed genes (P < 0.01) were determined by edgeR and colored in red. (PDF) [file pone.0148311.s003.pdf]
